# SGLT2 Inhibitors in Patients with Chronic Kidney Disease and Heart Disease: A Literature Review

**DOI:** 10.14797/mdcvj.1120

**Published:** 2022-09-06

**Authors:** Abhishek Kansara, Faiza Mubeen, Jawairia Shakil

**Affiliations:** 1 Houston Methodist Hospital, Houston, Texas, US; 2 Weill Cornell College of Medicine, Ithaca, New York, US; 3 Texas A&M College of Medicine, Houston, Texas, US

**Keywords:** SGLT2 inhibitors, cardiovascular outcomes, renal outcomes, heart failure, diabetic ketoacidosis, DKA

## Abstract

Sodium-glucose transport protein 2 inhibitors, commonly referred to as SGLT2i, are a group of prescription pharmaceuticals that are approved by the United States Food and Drug Administration for use with diet and exercise to lower blood glucose in adults with type 2 diabetes. Diabetes is a well-recognized major contributor to cardiovascular and renal disease burden. In addition to blood glucose control, SGLT2i have been shown to provide significant cardiovascular and renoprotective benefits in patients with and without diabetes. In this review, we describe current evidence related to the renal and cardiovascular benefits of using SGLT2i.

## Introduction

Sodium-glucose transporter 2 (SGLT2) is the major transport protein of the sodium-glucose transporter protein family and provides approximately 90% of glucose reabsorption from the kidneys.^1^ Inhibiting SGLT2 reduces blood glucose independently of insulin secretion and sensitivity. SGLT2 inhibitors (SGLT2i) are a class of pharmaceuticals that lower blood glucose by causing glucosuria, thereby inhibiting glucose reabsorption from glomerular filtration back into circulation.^2,3^ In addition, SGLT2i have been shown to provide significant cardiovascular and renal protection in patients with and without diabetes.^4^ In 2013, canagliflozin was the first SGLT2i approved by the United States (US) Food and Drug Administration (FDA) for the treatment of type 2 diabetes. Since then, several more SGLT2i have been approved (Table 1). Since their initial FDA approval for management of type 2 diabetes, SGLT2i have gained expanded approval for usage in conditions apart from diabetes. In this article, we note the evidence found in clinical trials that highlight the cardiovascular and renal benefits of this class of agents, especially with respect to heart failure and progression of kidney disease.^1^

**Table 1:** SGLT2i approved by the United States Food and Drug Administration. CV: cardiovascular; DKA: diabetic ketoacidosis; eGFR: estimated glomerular filtration rate; NYHA: New York Heart Association; CKD: chronic kidney disease.


SGLT2i	TRADE NAME	FDA APPROVAL	LIMITATIONS OF USE

**Canagliflozin** **Initial US** **approval: 2013**	**Invokana**	As an adjunct to diet and exercise to improve glycemic control in adults with type 2 diabetes mellitus To reduce the risk of major adverse cardiovascular events in adults with type 2 diabetes mellitus and established cardiovascular disease	Not for treatment of type1 diabetes mellitus or DKA

**Dapagliflozin** **Initial US** **approval: 2014**	**Farxiga**	To reduce the risk of sustained eGFR decline, end-stage kidney disease, CV death, and hospitalization for heart failure in adults with chronic kidney disease at risk of progression To reduce the risk of cardiovascular death and hospitalization for heart failure in adults with heart failure (NYHA class II-IV) with reduced ejection fraction To reduce the risk of hospitalization for heart failure in adults with type 2 diabetes mellitus and either established cardiovascular disease or multiple CV risk factors As an adjunct to diet and exercise to improve glycemic control in adults with type 2 diabetes mellitus	Not for treatment of type 1 diabetes mellitus; may increase the risk of DKA Not for use to improve glycemic control in adults with type 2 diabetes mellitus with an eGFR less than 45 mL/min/1.73 m^2^ Not for the treatment of CKD with polycystic kidney disease or patients requiring or with a recent history of immunosuppressive therapy for kidney disease

**Empagliflozin** **Initial US** **approval: 2014**	**Jardiance**	To reduce the risk of cardiovascular death and hospitalizationfor heart failure in adults with heart failure To reducethe risk of cardiovascular death in adults with type 2diabetes mellitus and established cardiovascular disease As an adjunct todiet and exercise to improve glycemic control in adults withtype 2 diabetes mellitus	Not for treatment of type 1diabetes mellitus; may increase the risk of DKA

**Ertugliflozin** **Initial US** **approval: 2017**	**Steglatro**	As an adjunct to diet and exercise to improve glycemic control in adults with type 2 diabetes mellitus	Not for treatment of type 1diabetes mellitus; may increase the risk of DKA


## SGLT2i in outcomes trials

### SGLT2i and Renal Outcomes

Several clinical trials have demonstrated the effects of SGLT2i on kidney disease, with effects on multiple kidney outcomes including albuminuria, doubling of serum creatinine, glomerular filtration rate (GFR) decline, kidney failure, and death.^5^ In the EMPA-REG trial, analysis of empagliflozin (an SGLT2i that received FDA approval in 2014) as a specific component of the secondary microvascular outcome decreased progression of renal disease in patients with type 2 diabetes mellitus (T2DM) and high cardiovascular risk compared to a placebo. The end points were worsening nephropathy, seen by doubling of serum creatinine levels, and initiation of renal replacement therapy, which demonstrated a significant relative risk reduction of 44% and 55%, respectively.^6^

The benefit of canagliflozin on the kidney was seen in the CREDENCE trial, which included patients with T2DM with macroalbuminuria (urinary albumin/creatinine ratio >300 mg/g and estimated GFR [eGFR] of 30–89 mL/min/1.73 m^2^). This trial reported a relative risk reduction of 30% in progression to end-stage renal disease, doubling of serum creatinine level, or mortality from renal or cardiovascular causes.^7^ The CANVAS trial showed a 25% risk reduction in the progression of albuminuria and a 40% risk reduction in the composite of 40% reduction in eGFR, renal replacement therapy, or renal death with the use of canagliflozin.^31^ The EMPEROR-REDUCED and VERTIS-CV trials, which studied heart failure and cardiovascular outcomes with empagliflozin and ertugliflozin, noted a similar risk reduction. The absolute difference in the eGFR in the empagliflozin group was higher by 1.73 mL/min/1.73m^2^ than in the placebo group,^33^ and in the ertugliflozin^48^ group was higher by 2.55 mL/min/1.73 m^2^ than in the placebo group.

When dapagliflozin was compared with placebo in patients with chronic kidney disease (CKD) with or without diabetes, the risk of eGFR decline of ≥ 50%, progression to end-stage renal disease, and renal or cardiovascular mortality was notably lower in the treatment group.^8^ The SCORED trial assessed cardiovascular outcomes in patients with diabetes and CKD (eGFR 25-60 mL/min/1,73 m^2^) with or without albuminuria. While the primary and secondary end points had to be modified due to a change in trial sponsorship and early closure, sotagliflozin^9^ failed to show any significant changes in long-term dialysis, renal transplantation, the first occurrence of a sustained decrease of ≥ 50% in eGFR, or sustained eGFR of < 15 mL/min/1.73 m^2^.

### SGLT2i in Kidney Transplant

While SGLT2i offer renoprotection, a looming question remains regarding their efficacy and safety in patients who have undergone kidney transplantation. A review of the literature shows that the antihyperglycemic effect of SGLT2i in patients with kidney transplant was comparable to the effect previously demonstrated in large randomized controlled trials in non-kidney transplant patients.^10,11,12^ A summary of kidney-related outcomes from major SGLT2i trials is represented in Table 2.

**Table 2:** Summary of renal outcomes trials of SGLT2 inhibitors. CKD: chronic kidney disease; eGFR: estimated glomerular filtration rate; ESRD: end-stage renal disease; RRR: relative risk reduction.


TRIAL	DRUG	NO. OF PARTICIPANTS	PERCENTAGE OF PARTICIPANTS WITH CKD, eGFR <60 ml/min/1.73m^2^	PRIMARY OUTCOME^#^	PROGRESSION OF ALBUMINURIA	COMPOSITE OF 40% REDUCTION IN eGFR, RENAL REPLACEMENT THERAPY OR RENAL DEATH	DOUBLING OF SERUM CREATININE	ESRD: eGFR <15, CHRONIC DIALYSIS, KIDNEY TRANSPLANTATION	DECLINE IN eGFR OF ≥50%	ABSOLUTE DIFFERENCE IN eGFR, (STUDY DRUG-PLACEBO, ml/min/1.73m^2^)	INCIDENT OR WORSENING NEPHROPATHY

**EMPA-REG OUTCOME 06/2016**	Empagliflozin	7,020	25.9	Table 3	38% RRR		44% RRR	46% RRR			39% RRR

**CANVAS 06/2017**	Canagliflozin	10,142	20.1	Table 3	27% RRR	40% RRR					

**CREDENCE 04/2019**	Canagliflozin	4,401	59.9	30% RRR			40% RRR	32% RRR			

**EMPEROR-REDUCED 08/2020**	Empagliflozin	3,730	48.2	Table 4		50% RRR				1.73 (1.1–2.37)	

**VERTIS-CV 09/2020**	Ertugliflozin	8,246	21.9	Table 3	21% RRR	34% RRR	NS			2.55 (1.5–3.61)	

**DAPA-CKD 09/2020**	Dapagliflozin	4,304	89.5	39% RRR				36% RRR	47% RRR		

**SCORED 11/2020**	Sotagliflozin	10,584	100	Table 3				NS*			


^#^ Primary outcomes varied between different studies. CREDENCE: Composite of end-stage kidney disease (dialysis, transplantation, eGFR < 15 ml/min/1.73m^2^), doubling of serum creatinine or death from renal or cardiovascular causes); DAPA-CKD: composite of first occurrence of any of decline of at least 50% in eGFR, onset of end-stage kidney disease (dialysis, kidney transplantation or eGFR < 15 ml/min/1.73m^2^), or death from renal or cardiovascular causes. ^*^ NS not significant for first occurrence of a sustained decrease of ≥50% in eGFR from baseline for ≥30 days, long-term dialysis, renal transplantation, or sustained eGFR of ≤15 ml/min/1.73m^2^ for ≥30 days.

### SGLT2i and Cardiovascular Outcomes

It is well established that diabetes is a major risk factor for cardiovascular disease (CVD), which affects about a third of individuals with T2DM. Additionally, at least half of the mortality in individuals with T2DM can be attributed to CVD.^13,14^ In recent years, several clinical trials have shown robust evidence regarding the benefits of SGLT2i with respect to cardiovascular outcomes. These studies have included patients with either preexisting heart disease or those with an elevated risk for heart disease. What is striking in these studies is that across different agents in this class, they show a uniform relative risk reduction in hospitalization for heart failure, and considerable heterogeneity exists when assessing the primary cardiovascular end point, which includes death from cardiovascular causes, nonfatal myocardial infarction, or nonfatal stroke. Only empagliflozin^15^ and canagliflozin^16^ have shown clinically significant benefits with regard to the primary outcome. Empagliflozin is the only agent to show significant risk reduction for death from cardiovascular or any cause. Ertugliflozin shows no clinically significant benefit in any of the primary or secondary outcomes studied, but it did show a statistically significant risk of hospitalization for heart failure (Table 3). Sotagliflozin also showed a 26% reduction in the modified primary end points. Importantly, as with most SGLT2i drug trials, the benefit in the primary outcomes was likely secondary to the reduction in hospitalization for heart failure.^9^ Table 3 shows a summary of cardiovascular outcome trials for SGLT2i.

**Table 3:** Summary of cardiovascular outcome trials of SGLT2i.


TRIAL	Drug	NO. OF PARTICI-PANTS	MEDIAN FOLLOW-UP, YEARS	MALE GENDER STUDY POPULATION, %, STUDY ARM/PLACEBO ARM	PRE-EXISTING CVD, %	PREEXISTING HEART FAILURE,%	CKD WITH eGFR <60 ml/min/1.73m^2^, %	PRIMARY OUTCOME^	HOSPITALIZATION FOR HEART FAILURE	DEATH FROM CV CAUSES	DEATH FROM ANY CAUSE

**EMPA-REG, 2015**	Empagliflozin	7020	3.1	71.2/72	>99	10.1	25.9	14% RRR	35% RRR	38% RRR	32% RRR

**CANVAS, 2017**	Canagliflozin	10,142	126.1 weeks	64.9/63.3	65.5	14.4	20.1	14% RRR	33% RRR	NS	NS

**DECLARE-TIMI 58, 2018**	Dapagliflozin	17,160	4.2	63.1/62.1	40.6	10.0	7	NS*	27% RRR	NS	NS

**VERTIS-CV, 2020**	Ertugliflozin	8,246	3.0	70.3/69.3	100	23.7	21.9	NS*	30% RRR	NS	NS

**SCORED, 2020**	Sotagliflozin	10,584	16 months	55.7/54.5	At least 22%	31%	100	26% RRR	33% RRR	NS	NS


^ Primary outcome = death from cardiovascular causes, nonfatal MI, or nonfatal stroke; RRR: relative risk reduction; NS: not significant, * significant for noninferiority.

### Heart Failure Outcomes

Further trials including patients with heart failure have helped to better delineate these outcomes. Empagliflozin showed an overall risk reduction in the primary outcomes of cardiovascular death or hospitalization for heart failure of anywhere from 21% to 25% in patients with either heart failure with preserved ejection fraction (HFpEF) or with reduced ejection fraction (HFrEF).^17,18^ Dapagliflozin showed similar outcomes in patients with HFrEF, with a 26% overall risk reduction.^19^ Although a study of sotagliflozin ended early due to lack of funding from the sponsor, it showed a 33% risk reduction with a median follow-up of only 9 months.^20^ Moreover, the CANVAS program reduced the overall risk of heart failure events but had no clear difference in effects on patients with HFrEF versus HFpEF. It is important to recognize that in these studies, no death benefit was noted except in the dapagliflozin study.

A meta-analysis of more than 20,000 patients from 15 randomized controlled trials noted a 31% reduction in hospitalization for heart failure and a 61% reduction in urgent visits for heart failure.^21^ Additionally, Cardoso et al. noted a 14% reduction in all-cause mortality and a similar reduction in cardiovascular mortality. These findings were significantly lower in individuals treated with SGLT2i across subgroups of age, sex, race, renal function, and heart failure functional classification. Another meta-analysis by McGuire et al. studied over 46,000 patients from six trials and noted a 32% risk reduction in hospitalization for heart failure and, despite significant heterogeneity of associations with outcomes, a 15% risk reduction for cardiovascular death.^22^

These studies and meta-analyses may indicate an improvement in quality of life and functional status, due to a uniform risk reduction in hospitalization for heart failure, whereas mortality benefits show only some heterogeneity among studies. Additional studies with dapagliflozin and canagliflozin have assessed and reported quality of life outcomes. The PRESERVED-HF trial assessed the primary end point of Kansas City Cardiomyopathy Questionnaire Clinical Summary Score (KCCQ-CS) and 6-minute walk test (6MWT) in 324 patients with HFpEF after 12 weeks of treatment with dapagliflozin.^23^ The study reported a 5.8-point improvement in KCCQ-CS and an improvement of 20.1 meters in the 6MWT, both statistically significant, while noticing reduced weight, but no differences were reported in the N-terminal pro–B-type natriuretic peptide (NT-proBNP), BNP levels, or other secondary end points. The CHIEF-HF trial, which was conducted entirely virtually, assessed the primary outcome of change in the Kansas City Cardiomyopathy Questionnaire Total Symptom Score at 12 weeks in 476 participants who were randomized to either canagliflozin or placebo.^24^ The change in score was 4.3 points higher with canagliflozin than with placebo, independent of ejection fraction or diabetes status. While this study has its limitations due to being virtual, the improvement in the clinical outcome is significant and may be extrapolated more widely (Table 4).

**Table 4:** Summary of heart failure outcomes trials of SGLT2i. CVD: cardiovascular disease; CKD: chronic kidney disease; eGFR: estimate glomerular filtration rate; RRR: relative risk reduction; NS: not significant.


TRIAL	DRUG	NO. OF PARTICIPANTS	MEDIAN FOLLOW-UP	MALE GENDER STUDY POPULATION, %, STUDY ARM/PLACEBO ARM	PREEXIST-ING CVD, %	PREEXISTING HEART FAILURE, %	CKD WITH eGFR <60 ml/min/1.73 m^2^, %	PRIMARY OUTCOME^^	HOSPITALIZATION FOR HEART FAILURE	DEATH FROM CV CAUSES	DEATH FROM ANY CAUSE

**DAPA-HF, 2019**	Dapagliflozin	4,744	18.2 months	76.2/77	56.4^#^	100	40.6	26% RRR	30% RRR	18% RRR	17%

**EMPEROR-Reduced, 2020**	Empagliflozin	3,730	16 months	76.5/75.6	51.8^#^	100	48.2	25% RRR	31% RRR	NS	NS

**EMPEROR-Preserved, 2021**	Empagliflozin	5,988	26.2 months	55.4/55.3	35.4^#^	100	49.9	21% RRR	29% RRR	NS	NS

**SOLOIST-WHF, 2020**	Sotagliflozin	1,222	9.0 months	67.4/65.1	58.3^#^	100	NA	33% RRR	36% RRR	NS	NS


^^ Primary outcome = composite of adjudicated CV death or worsening/hospitalization for heart failure; #: ischemic cardiomyopathy.

These studies indicate that SGLT2i not only improve cardiac outcomes as measured in clinical trials but likely contribute to an improved quality of life, with improved walk scores and fewer symptoms associated with heart failure.

## Effects of SGLT2i apart from glucosuria

### Renal Effects

SGLT2i help to restore the tubulo-glomerular feedback by increasing sodium chloride transport to macula densa, which causes the release of vasoactive substances including adenosine.^25,26^ Adenosine locally binds to receptors, causing afferent arteriole constriction; this leads to decreases in glomerular perfusion and filtration, which otherwise cause progressive glomerular injury.^27,28^ In contrast, a study in patients with T2DM also shows that SGLT2i causes efferent arteriole dilatation, which reduces GFR.^29^ Proximal tubular cells require high oxygen consumption for energy and adenosine triphosphate (ATP)-dependent reabsorption of electrolytes and other organic molecules. Glucose reabsorption via SGLT2 increases in patients with T2DM due to hyperglycemia, which results in hyperfiltration and increased luminal glucose load.^30^ As a result, the oxygen demand of tubular cells increases, leaving the proximal tubule relatively hypoxic.^31^ SGLT2i decrease the reabsorption of sodium and glucose, thus reducing proximal tubular workload and overcoming the hypoxia.

In early experimental and human diabetes studies, glucose metabolic flux increases in the kidney cortex due to increased glucose uptake in proximal tubular cells, which can lead to mitochondrial dysfunction.^32^ SGLT2 inhibition reduces progression of mitochondrial dysfunction and alleviates hypoxia and ATP depletion. In addition, the favorable effect of SGLT2i versus a diuretic in promoting fluid shift, especially from the renal interstitium, and decreased energy consumption by proximal tubular cells may alleviate cortical and outer medullary hypoxia.^33^ Alternate mechanisms of action may include increased circulating ketones,^34^ causing a shift from glucose to lipid oxidation that, together with an increase in the glucagon-to-insulin ratio, provide increased ketone production, improved myocardial energetics, improved ionic hemostasis, altered adipokine regulation, and autophagy.

### Cardiovascular Effects

Although the causes for heterogeneity in several cardiovascular outcomes remain unexplained, a common theme across this family of hypoglycemic pharmacotherapy is its significant benefit in hospitalization for heart failure. Given that benefits to heart failure hospitalization have been noted rather early in the studies, the effects are likely not associated with the glucose-lowering properties and changes with blood pressure and cholesterol due to SGLT2i therapy, which often take a long time to yield any beneficial effects. Additionally, these benefits are noted in patients with or without diabetes and irrespective of ejection fraction and across a spectrum of renal function. SGLT2i cause glucosuria and natriuresis, which facilitate improvement in ventricular loading by reducing preload; ensuing osmotic diuresis also contributes.^35^ It is theorized that SGLT2i may influence cardiac energy metabolism by shifting cardiac energy reliance from non-esterified fatty acids and glucose to ketones; ketones may increase the mechanical efficiency of the failing heart and are considered a “super fuel” for the failing myocardium. Another interesting hypothesis is that inhibition of Na+/H+ exchanger (NHE) 1 and NHE3 by SGLT2i in both the kidney and the heart may be a common mechanism through which these agents offer cardioprotection and renoprotection. Some experimental models and preliminary studies have shown antifibrotic properties of dapagliflozin and empagliflozin, respctively.^36,37^ It has been suggested that SGLT2i may restore the inflammatory adipokine balance, but this theory has not been confirmed. Though the exact process remains unknown, it has been observed that SGLT2i decrease inflammatory markers, including nuclear factor-kB, interleukin 6 (IL-6), monocyte chemoattractant protein 1 (MCP-1), and many other factors in investigational models of diabetes.^38,39^ Similarly, a decrease in serum tumor necrosis factor receptor 1, IL-6, urine IL-6, and MCP-1 was seen in patients with T2DM on SGLT2i.^40,41^ SGLT2i also reduced uric acid levels in patients with T2DM, thus reducing the inflammatory effects of uric acid that contribute to diabetic kidney disease.^42,43^ Joshi et al. highlighted the potential mechanisms by classifying them as conventional and novel.^44^ The understood conventional mechanisms can be described as diuresis and blood pressure reduction, weight loss, blood glucose control, and hemoconcentration, while the novel mechanisms comprise the improved myocardial energetics, ionic hemostasis, autophagy, and altered adipokine regulation described above.

## The role of SGLT2i in primary prevention

### Effect on Lipids

A meta-analysis of 48 randomized controlled trials revealed that SGLT2i significantly increased total cholesterol, low-density lipoprotein (LDL)-cholesterol, high-density lipoprotein (HDL)-cholesterol, and non-HDL-cholesterol.^45^ Additionally, SGLT2i administration significantly decreased triglyceride levels. No significant alteration in LDL/HDL ratio was found after SGLT2i treatment. The dapagliflozin trial demonstrated significant benefit of raising HDL cholesterol level for improving reverse cholesterol transport.^46^ Overall, the effects of SGLT2i on dyslipidemia in both human and animal studies are inconclusive.

### Effect on Weight

SGLT2i promote weight loss via glucosuria (average 70 grams of glucose per day or 270 kcal) and negative energy balance, leading to significant weight loss.^47^ SGLT2i use is associated with a reduction of major adverse cardiovascular events through clinically significant weight loss.^48^ Compared with placebo, results of network meta-analyses show reductions of body weight for all SGLT2i treatments of about 1.5 kg to 2 kg,^49,50^ depending on the dose.^51,52^

### Prevention of Diabetes

A prespecified pooled analysis of the DAPA-CKD and DAPA-HF trials showed that dapagliflozin reduced the incidence of new-onset diabetes by approximately one-third.^53^ Among people with prediabetes, the number needed to treat was 50 patients per year to prevent one case of new-onset diabetes (absolute risk reduction of 2 events per 100 patient-years). Several mechanisms have been proposed, including protection of pancreatic beta cells from glucose toxicity. Indirect mechanisms could include decrease in insulin resistance through weight loss and improvement of hepatic insulin sensitivity. SGLT2i may thus play a role in the primary prevention of cardiovascular and kidney disease.

## SGLT2i safety concerns and adverse effects

Severe adverse effects of SGLT2i are rare but have been reported through clinical trials and post-marketing data, as highlighted below.

### Hypoglycemia

SGLT2i used as a monotherapy are all associated with a low risk of developing hypoglycemia. However, the risk is higher when SGLT2i are utilized as an add-on therapy with sulfonylurea or insulin.^54^

### Mycotic Infections of Perineum and Urinary Tract Infections (UTIs)

The risk for genital candidiasis increases with the development of glycosuria in poorly controlled hyperglycemia. The most common adverse event of SGLT2i as a group, compared with placebo groups, is mycotic infections, particularly among females.^55^ A metanalysis found that the risk of mycotic genital infections is lower when SGLT2i is combined with dipeptidyl peptidase-4 (DPP-4) inhibitors than when they are used as a monotherapy or as an adjunct to metformin.^56^ Necrotizing fasciitis of the perineum, called Fournier’s gangrene, is a significant adverse event, although post-marketing case reviews show a lower incidence than initially reported.^57^

### Diabetic Ketoacidosis and Euglycemic Diabetic Ketoacidosis

The use of SGLT2i has been associated with an increased risk of diabetic ketoacidosis (DKA). The rates of DKA in major trials were 0.1% to 0.5% over 4 to 8 years.^58^ The FDA and European Medicines Agency issued safety reports on DKA risk in patients treated with SGLT2i who showed DKA-like symptoms.^59^ Considering the insulin-independent inhibition of glycemia, DKA may present with mildly elevated or even normal blood glucose levels, leading to delayed diagnosis and management under the term euglycemic DKA (EDKA).^60^ The risk of EDKA is significantly higher in the clinical settings of acute illness, fasting, perioperative states, or excess alcohol consumption.

### Amputation

In the CANVAS clinical trial, canagliflozin showed an increased risk of toe and foot amputation. The higher risk of amputation seems inconsistent, as it was reported only in the CANVAS trial but not in empagliflozin or dapagliflozin trials, nor in the subsequent CREDENCE trial with canagliflozin.^61^ However, the true risk of this complication as a causation or even a consistent correlation is not clear.^62^

### Fractures

SGLT2i are associated with a small increase in fractures, as noted in the CANVAS study thus far,^31^ but other SGLT2i randomized clinical trials and meta-analyses have failed to show an increase in fractures. A recent study based on real-world, population-based Medicare data noted no increase in risk of fractures in patients treated with SGLT2i compared to patients treated with either DPP-4 or glucagon-like peptide-1 receptor agonists.^63^ However, it is expected to be an uncertain complication in future trials.^64^

### Use in Chronic Kidney Disease/Risk of Acute Kidney Injury

Data on using SGLT2i in severe or end-stage renal failure are scarce. Some studies have suggested that SGLT2i may prevent the development of acute kidney injury (AKI) despite adverse events related to hypovolemia.^64,65^ The mechanism may be reduced renal hyperfiltration, though an initial and transient decline in eGFR usually follows SGLT2i commencement. In summary, SGLT2i is clinically safe to use in patients with diabetes who have mild to moderate renal failure. The safety of use in patients with eGFR less than 30 is less clear.

## Conclusion

Although SGLT2i were developed as antihyperglycemic agents, a growing body of evidence demonstrates consistent reductions in risks for secondary kidney disease end points (albuminuria and a composite of serum creatinine doubling or eGFR decline, kidney failure, or death) and reductions in CVD events. Although not as robust, there are emerging data on using SGLT2i in patients who have received a kidney transplant. In addition to glycemic benefits, nonglycemic mechanisms are involved in CKD and CVD risk reductions. These effects improve glomerular hemodynamics, modify volume status, and modify local and systemic mechanisms involved in the pathogenesis of CKD and CVD. Due to significant benefits seen, use of SGLT2i in appropriate patient populations is encouraged. With the increasing use of these agents in patients with non-diabetes CVD (particularly heart failure) and CKD to prevent progression, it is of the utmost importance to identify potential significant adverse effects, such as EDKA and genitourinary infections. Although not common, these adverse effects can lead to significant morbidity and in some cases mortality. Therefore, efforts to disseminate awareness and recognition of these adverse effects is imperative since the increased use of these agents is expected to continue.

## Key points

- Sodium-glucose transporter 2 inhibitors (SGLT2i), a group of medications that were initially approved to treat type 2 diabetes, are increasingly used for indications such as heart failure and chronic kidney disease prevention in patients without diabetes.
- There are significant data regarding the cardiovascular and renal benefits of these medications in patients with or without diabetes, such as decreased progression of renal disease in patients with type 2 diabetes and high cardiovascular risk, and decreased risk of cardiovascular death or hospitalization for heart failure.
- Significant adverse effects have been shown in clinical trials and are seen in clinical practice. Of particular concern are euglycemic diabetic ketoacidosis (DKA), genitourinary infections including severe infections, and reports of limb amputations.
- Clinicians need to be aware of potential adverse effects of using SGLT2i in patients who may be at high risk of infection, DKA, or limb amputation.
